# Analysis of Biased Competition and Cooperation for Attention in the Cerebral Cortex

**DOI:** 10.3389/fncom.2019.00051

**Published:** 2019-07-31

**Authors:** Tatyana Turova, Edmund T. Rolls

**Affiliations:** ^1^Mathematical Center, University of Lund, Lund, Sweden; ^2^IMPB - The Keldysh Institute of Applied Mathematics of the Russian Academy of Sciences, Moscow, Russia; ^3^Oxford Centre for Computational Neuroscience, Oxford, United Kingdom; ^4^Department of Computer Science, University of Warwick, Coventry, United Kingdom; ^5^Institute of Science and Technology for Brain-Inspired Intelligence, Fudan University, Shanghai, China

**Keywords:** biased competition, attention, cerebral cortex, mathematical analysis, neural networks, top-down connections, bottom-up connections

## Abstract

A new approach to understanding the interaction between cortical areas is provided by a mathematical analysis of biased competition, which describes many interactions between cortical areas, including those involved in top-down attention. The analysis helps to elucidate the principles of operation of such cortical systems, and in particular the parameter values within which biased competition operates. The analytic results are supported by simulations that illustrate the operation of the system with parameters selected from the analysis. The findings provide a detailed mathematical analysis of the operation of these neural systems with nodes connected by feedforward (bottom-up) and feedback (top-down) connections. The analysis provides the critical value of the top-down attentional bias that enables biased competition to operate for a range of input values to the network, and derives this as a function of all the parameters in the model. The critical value of the top-down bias depends linearly on the value of the other inputs, but the coefficients in the function reveal non-linear relations between the remaining parameters. The results provide reasons why the backprojections should not be very much weaker than the forward connections between two cortical areas. The major advantage of the analytical approach is that it discloses relations between all the parameters of the model.

## 1. Introduction

Biological systems employ a selection processing strategy for managing the enormous amount of information resulting from their interaction with the environment. This selection of relevant information is referred to as attention. One type of visual attention is the result of top-down influences on the processing of sensory information in the visual cortex, and therefore is intrinsically associated with neural interactions within and between cortical areas. Thus, elucidating the neural basis of visual attention is an excellent paradigm for understanding some of the basic mechanisms for interactions between cortical areas.

Observations from a number of cognitive neuroscience experiments have led to an account of attention termed the “*biased competition hypothesis*,” which aims to explain the computational processes governing visual attention and their implementation in the brain's neural circuits and neural systems. According to this hypothesis, attentional selection operates in parallel by biasing an underlying competitive interaction between multiple stimuli in the visual field toward one stimulus or another, so that behaviorally relevant stimuli are processed in the cortex while irrelevant stimuli are filtered out (Chelazzi et al., 1993; Duncan, 1996; Chelazzi, 1998; Reynolds and Desimone, 1999). Thus, attending to a stimulus at a particular location or with a particular feature biases the underlying neural competition in a certain brain area in favor of neurons that respond to the location, or the features, of the attended stimulus. This attentional effect is produced by generating signals in areas outside the visual cortex which are then fed back to extrastriate visual cortical areas, where they bias the competition such that when multiple stimuli appear in the visual field, the cells representing the attended stimulus *win*, thereby suppressing the firing of cells representing distracting stimuli (Duncan and Humphreys, 1989; Desimone and Duncan, 1995; Duncan, 1996; Reynolds et al., 1999). According to this line of work, attention appears as a property of competitive/cooperative interactions that work in parallel across the cortical modules. Neurophysiological experiments are consistent with this hypothesis in showing that attention serves to modulate the suppressive interaction between the neuronal firing elicited by two or more stimuli within the receptive field (Miller et al., 1993; Motter, 1993; Chelazzi, 1998; Reynolds and Desimone, 1999; Reynolds et al., 1999). Further evidence comes from functional magnetic resonance imaging (fMRI) in humans (Kastner et al., 1998, 1999) which indicates that when multiple stimuli are present simultaneously in the visual field, their cortical representations within the object recognition pathway interact in a competitive, suppressive fashion, which is not the case when the stimuli are presented sequentially. It was also observed that directing attention to one of the stimuli counteracts the suppressive influence of nearby stimuli.

Neurodynamical models providing a theoretical framework for biased competition have been proposed and successfully applied in the context of attention and working memory (Rolls and Deco, 2002; Rolls, 2016). In the context of attention, Usher and Niebur (1996) introduced an early model of biased competition to explain the attentional effects in neural responses observed in the inferior temporal cortex, and this was followed by a model for V2 and V4 by Reynolds et al. (1999) based on the shunting equations of Grossberg (1988). Deco and Zihl (2001) extended Usher and Niebur's model to simulate the psychophysics of visual attention by visual search experiments in humans. Their neurodynamical formulation is a large-scale hierarchical model of the visual cortex whose global dynamics is based on biased competition mechanisms at the neural level. Attention then appears as an effect related to the dynamical evolution of the whole network. This large-scale formulation has been able to simulate and explain in a unifying framework visual attention in a variety of tasks and at different cognitive neuroscience experimental measurement levels, namely: single-cells (Deco and Lee, 2002; Rolls and Deco, 2002), fMRI (Corchs and Deco, 2002, 2004), psychophysics (Deco et al., 2002; Deco and Rolls, 2004), and neuropsychology (Deco and Rolls, 2002; Heinke et al., 2002). In the context of working memory, further developments (Deco and Rolls, 2003; Deco et al., 2004; Szabo et al., 2004) managed to model in a unifying form attentional and memory effects in the prefrontal cortex integrating single-cell and fMRI data, and different paradigms in the framework of biased competition.

A detailed dynamical analysis of the synaptic and spiking mechanisms underlying biased competition was produced by Deco and Rolls (2005). However, the parameter regions within which biased competition operates were identified by a mean field analysis, which consisted of testing a set of parameters until effective regions in the state spaces were identified.

Here we treat the biased competition system analytically for the first time. This mathematical analysis complements previous numerical results and improves our understanding of the principles of operation of the system. Although the results are presented in the context of attention, they apply more generally to interactions between cortical areas. The dynamics of cortical attractor networks, and the dynamical interactions between cortical areas and the strength of the connections between them that enable them to interact usefully for short-term memory and attention yet maintain separate attractors have been analyzed with the rather different approaches of theoretical physics elsewhere (Treves, 1993; Battaglia and Treves, 1998; Renart et al., 1999a,b, 2000, 2001; Panzeri et al., 2001; Rolls, 2016).

## 2. Methods

### 2.1. The Biased Competition Network

**Figure 1:**
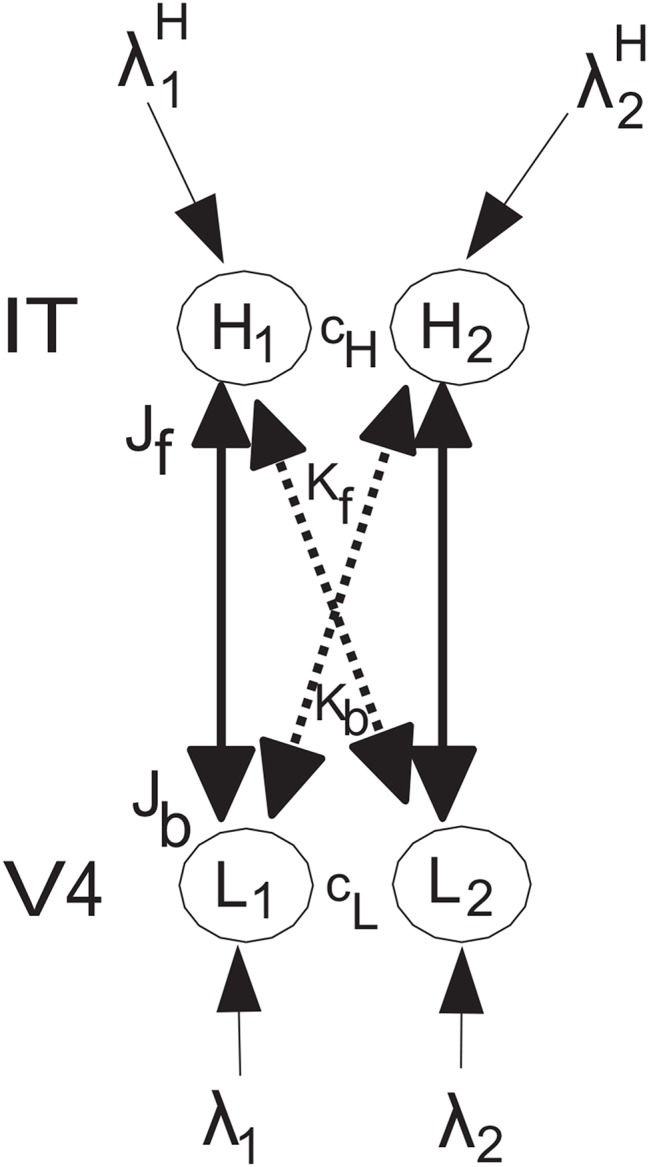
Model architecture. The lower nodes are *L*_1_ and *L*_2_, and receive inputs λ_1_ and λ_2_. *L*_1_ has forward connections of strength *J*_*f*_ to higher node *H*_1_ for which stimulus 1 acting via λ_1_ is the preferred input, and *L*_2_ has forward connections of strength *J*_*f*_ to higher node *H*_2_ for which stimulus 2 acting via λ_2_ is the preferred input. The corresponding backprojections have weaker strength *J*_*b*_. In addition, there are crossed connections shown with dashed lines. In particular, *L*_1_ has forward connections of weak strength *K*_*f*_ to higher node *H*_2_ as stimulus 1 is not the preferred stimulus for *H*_2_, and *L*_2_ has forward connections of strength *K*_*f*_ to higher node *H*_1_ as stimulus 2 is not the preferred stimulus for *H*_1_. The corresponding backprojections have weaker strength *K*_*b*_. A top-down attentional bias signal $\lambda_{2}^{H}$ can be applied to node *H*_2_, and can bias the network to emphasize the effects of λ_2_ even if λ_2_ is less than or equal to λ_1_. There is competition within a level, with *H*_1_ and *H*_2_ competing with strength *c*_*H*_, and *L*_1_ and *L*_2_ competing with strength *c*_*L*_. The conditions for these effects to occur and for the network to be stable are analyzed. In addition, all four of the nodes can in the simulations have recurrent collateral connections that produce self-excitatory effects, consistent with cortical architecture, and these produce the expected effects, but are not considered further here because the mathematical analysis focusses on the tractable case in which they are not present. The nodes *L* correspond to V4, and the nodes *H* to inferior temporal visual cortex, in the neurophysiological experiment of Chelazzi et al. (1993) on object-based top-down attention.

The network to be analyzed is shown in Figure 1, and has the same general architecture used by Deco and Rolls (2005) to investigate the mechanisms of biased competition in a range of neurophysiological experiments. The system has forward connections *J*_*f*_ and *K*_*f*_, and top-down backprojection connections *J*_*b*_ and *K*_*b*_, as shown in Figure 1. The top-down connections are weaker, so that they can bias the bottom up inputs, but not dominate them, so that the system remains driven by the world. The top-down connections in the model correspond to the backprojections found between adjacent cortical areas in a cortical hierarchy, and in the area of memory recall, to the backprojections from the hippocampus to the neocortex (Kesner and Rolls, 2015; Rolls, 2016, 2018). The anatomical arrangement that facilitates this is that the backprojections end on the apical dendrites of cortical pyramidal cells far from the cell body, where their effects can be shunted by forward inputs that terminate on the parts of the dendrite that are electrically closer to the cell body (Rolls, 2016). In both top-down attention, and in memory recall, it is important that any bottom-up inputs from the world take priority, so that the organism is sensitive to events in the world, rather than being dominated by internal processing (Rolls, 2016). Interestingly, there is evidence that this situation is less the case in schizophrenia, in which some key forward connections are reduced in magnitude relative to the backprojections (Rolls et al., 2019). The type of neurophysiological experiment for which this model was designed is described by Deco and Rolls (2005), with one of the original neurophysiological investigations performed on object-based attention in the inferior temporal visual cortex (IT) by Chelazzi et al. (1993). The overall operation of this architecture is conceptualized as follows [see Deco and Rolls (2005)]. Activity in neuron or population of neurons *L*_1_ have a strong driving effect on *H*_1_ (via *J*_*f*_), as stimulus 1 acting via λ_1_ is the preferred (i.e., most effective) stimulus, and a weaker effect on *H*_2_ (via the crossed connection *K*_*f*_) for which stimulus 1 is the less preferred stimulus. There are weaker corresponding backprojections *J*_*b*_ and *K*_*b*_. Correspondingly, *L*_2_ has a strong driving effect on *H*_2_ (via *J*_*f*_) as stimulus 2 acting via λ_2_ is the preferred stimulus, and a weaker effect on *H*_1_ (via the crossed connection *K*_*f*_) for which stimulus 2 is the less preferred stimulus. *L*_1_ is in competition with *L*_2_ (with strength *c*_*L*_), and *H*_1_ is in competition with *H*_2_ (with strength *c*_*H*_). The present model does not imply that *L*_1_ and *L*_2_ are in different areas in a topographical map, but that can be easily implemented by decreasing the strength of *c*_*L*_ if *L*_1_ and *L*_2_ are not close together in the map, and the same general results hold (see Deco and Rolls, 2005). It is noted that convergence in topographically mapped systems from stage to stage is important in providing one of the bases for translation invariant visual object recognition as modeled in VisNet (Rolls, 2012, 2016), and that the dendritic morphology at different stages of processing in the visual cortical hierarchy may facilitate this (Elston, 2002; Elston and Fujita, 2014).

The nodes *L* correspond to V4, and the nodes *H* to inferior temporal visual cortex, in the neurophysiological experiment of Chelazzi et al. (1993) on object-based top-down attention. The lower nodes are *L*_1_ and *L*_2_, and receive inputs λ_1_ and λ_2_. *L*_1_ has forward connections of strength *J*_*f*_ to higher node *H*_1_, and *L*_2_ has forward connections of strength *J*_*f*_ to higher node *H*_2_. The corresponding backprojections have strength *J*_*b*_. In addition, there are crossed connections *K*_*f*_ and *K*_*b*_ shown with dashed lines. The rationale for this connectivity is that the preferred stimulus for *L*_1_ has strong effects on *H*_1_, but weaker effects on *H*_2_ for which it is not the preferred stimulus. In a corresponding way, the preferred stimulus for *L*_2_ has strong effects on *H*_2_, but weaker effects on *H*_1_ for which it is not the preferred stimulus. This is implemented as follows. *L*_1_ has forward connections of strength *K*_*f*_ to higher node *H*_2_, and *L*_2_ has forward connections of strength *K*_*f*_ to higher node *H*_1_. The corresponding backprojections have strength *K*_*b*_. The *K* connections are weaker that the *J* connections. A top-down attentional bias signal $\lambda_{2}^{H}$ can be applied to node *H*_2_, and can bias the network to emphasize the effects of λ_2_ even if λ_2_ is less than or equal to λ_1_. There is competition within a level, with *H*_1_ and *H*_2_ competing with strength *c*_*H*_, and *L*_1_ and *L*_2_ competing with strength *c*_*L*_. The conditions for these effects to occur and for the network to be stable are analyzed. In addition, all four of the nodes can have recurrent collateral connections that produce self-excitatory effects, but the effects of these in the simulations do not affect the generic results obtained, and are not included for simplicity in the mathematical analyses.

The network can operate as follows. If a weaker than λ_1_ input λ_2_ is applied to *L*_2_, then neural populations *L*_1_ and *H*_1_ win the competition. However, if a top-down biased competition input $\lambda_{2}^{H}$ is applied to *H*_2_, then this can bias the network so that *H*_2_ wins the competition over *H*_1_, but also *L*_2_ has higher activity than *L*_1_. Here we analyse the relation between the parameters shown in Figure 1 that enable these effects to emerge and to be stable.

A feature of the model described here and also by Deco and Rolls (2005) is that local attractor dynamics are implemented within each of the neuronal populations, as recurrent collateral connections are a feature of the cerebral neocortex, and are one way to incorporate non-linear effects into the operation of the system. The new results are derived here analytically, and are illustrated with simulations with the parameters originating from the formulas obtained.

### 2.2. The Attentional Effects to be Modeled: Investigation 1

One top-down biased competition effect to be considered is as follows. If λ_1_ is greater than λ_2_, then the activity of *L*_1_ will be greater than the activity of *L*_2_. But if we apply a top-down biased competition input $\lambda_{2}^{H}$ to *H*_2_, then at some value of $\lambda_{2}^{H}$ the top-down bias will result in *L*_2_ having as much activity as *L*_1_. We seek to analyse the exact conditions under which this biased competition effect occurs, in terms of all the parameters of the system.

This system has been studied previously as follows, but with a mean-field analysis to search the parameter space, rather than the analytic approach described here. Figure 1 shows a design associated with a prediction that can be made by setting the contrast-attention interaction study in the framework of the experimental biased competition design of Chelazzi et al. (1993) involving object attention. Deco and Rolls (2005) modeled this experiment by measuring neuronal responses from neurons in neuronal population (or pool) *H*_1_ in the inferior temporal cortex (IT) to a preferred and a non-preferred stimulus simultaneously presented within the receptive field. They manipulated the contrast or efficacy of the stimulus that was non-preferred for the neurons *H*_1_. They analyzed the effects of this manipulation for two conditions, namely without object attention, or with top-down object attention on the non-preferred stimulus 2 that produced input λ_2_, implemented by adding an extra bias $\lambda_{2}^{H}$ to *H*_2_. In the previous integrate-and-fire simulations (Deco and Rolls, 2005), top-down biased competition was demonstrated, and it was found that the attentional suppressive effect implemented through $\lambda_{2}^{H}$ on the responses of neurons *H*_1_ to the competing non-preferred stimulus (2) was higher when the contrast of the non-preferred stimulus (2) was at intermediate values. However, the operation of this type of network was not examined analytically, which is the aim of the present investigation.

### 2.3. The Attentional Effects to be Modeled: Investigation 2

A second top-down competition effect might be considered as follows. If λ_1_ is greater than λ_2_ and both are applied simultaneously, then the activity of *H*_1_ will be greater than the activity of *H*_2_. However, if we apply top-down bias $\lambda_{2}^{H}$ to *H*_2_, then we can influence the activity of *H*_2_ and *H*_1_ (through all the connections in the system) until *H*_2_ and *H*_1_ have the same activity (and at the same time there will be an effect on *L*_1_ and *L*_2_). We wish to quantify these effects analytically in terms of all the parameters of the system.

## 3. Dynamics

We shall use the notation that [*x*]^+^ = max{*x*, 0} for any rational value of *x*. We assume that *L*_1_(*t*), *L*_2_(*t*), *H*_1_(*t*), *H*_2_(*t*) are defined by the following system of recurrent equations in discrete time *t* = 0, 1, …:

(3.1) $$\begin{array}{l} L_{i}(\text{t}+1)=[L_{i}(t)+\lambda_{i}(t)+\sum_{k=1,2}w_{ik}^{b}H_{k}(t)-c_{L}L_{j}(t)-\beta_{L}L_{i}(t)\\ \;{+I\{L_{i}(t)>T_{L}\}(T_{L}-\alpha_{L}L_{i}(t))]}^{+}, \end{array}$$

(3.2) $$\begin{array}{l} H_{i}(t+1)=[H_{i}(t)+\lambda_{i}^{H}(t)+\sum_{k=1,2}w_{ik}^{f}L_{k}(t)-c_{H}H_{j}(t)-\beta_{H}H_{i}(t)\\ \;{+I\{H_{i}(t)>T_{H}\}(T_{H}-\alpha_{H}H_{i}(t))]}^{+}, \end{array}$$

$$\begin{array}{l} i=1,2,\;i\neq j. \end{array}$$

One can also use here any time step *t* = 0, τ, 2τ,…, treating τ as another parameter. Here **I** denotes the indicator function, i.e.,

$$I\{H_{i}(t)>T_{H}\}=\{\begin{array}{ll} 1, & \text{ if }H_{i}(t)>T_{H},\\ 0, & \text{ otherwise, } \end{array}$$

and similarly for **I**{*L*_*i*_(*t*) > *T*_*L*_}. This means that the terms in (3.1) and (3.2) which involve these indicator functions for the recurrent dynamics are present only if the activity in *L*_*i*_(*t*) or *H*_*i*_(*t*) is greater than the corresponding threshold *T*_*L*_ or *T*_*H*_. In the analysis described here, we assumed that *T*_*L*_ and *T*_*H*_ were infinity, so that the recurrent dynamics were not in operation, but the recurrent dynamics were tested in the simulations.

The constants $w_{ij}^{f}$ represent the synaptic weight of the connection from node *L*_*j*_ to node *H*_*i*_, while $w_{ij}^{b}$ is the synaptic weight of the connection from node *H*_*j*_ to node *L*_*i*_. We shall assume that

$$\begin{array}{l} w_{ii}^{b}=J_{b},\;w_{ij}^{b}=K_{b},\;w_{ii}^{f}=J_{f},\;w_{ij}^{f}=K_{f}, \end{array}$$

for all *i* = 1, 2, *i* ≠ *j*, where indexes *b* and *f* correspond to “back” and “forward”. Furthermore, we assume that for some non-negative coefficient *q* (typically 0 ≤ *q* < 1, see more comments below)

(3.3) $$\begin{array}{l} J_{b}\geq K_{b},\;J_{f}\geq K_{f},\text{and}J_{b}=qJ_{f},\;K_{b}=qK_{f}. \end{array}$$

Functions $\lambda_{i}\left(t\right),\lambda_{i}^{H}\left(t\right)$ represent the external inputs.

All the remaining constants in the system (3.1), (3.2) are free parameters; they are assumed to be non-negative, as they represent the following characteristics:

β_*L*_ is the decay term for the *L* nodes,

*c*_*L*_ is the competition between the *L* nodes,

*T*_*L*_ is the threshold at which an *L* node enters its attractor dynamics as was explained above,

α_*L*_ is the gain factor for self-excitation in an *L* node for attractor dynamics,

β_*H*_ is the decay term for the *H* nodes,

*c*_*H*_ is the competition between the *H* nodes,

*T*_*H*_ is the threshold at which an *H* node enters its attractor dynamics,

α_*H*_ is the gain factor for self-excitation in an *H* node for attractor dynamics.

Note that when all the constant parameters of the system are set to be zero, including the input functions $\lambda_{i},\lambda_{i}^{H}$, then the system

(3.4) $$\begin{array}{l} \left(L_{1}\left(t\right),L_{2}\left(t\right),H_{1}\left(t\right),H_{2}\left(t\right)\right) \end{array}$$

remains at the initial state (*L*_1_(0), *L*_2_(0), *H*_1_(0), *H*_2_(0)).

We shall assume that

(3.5) $$\begin{array}{l} \left(L_{1}\left(0\right),L_{2}\left(0\right),H_{1}\left(0\right),H_{2}\left(0\right)\right)=\left(0,0,0,0\right), \end{array}$$

and that all the input functions are non-negative constants, i.e.,

(3.6) $$(\lambda_{1}(t),\lambda_{2}(t),\lambda_{1}^{H}(t),\lambda_{2}^{H}(t))=(\lambda_{1},\lambda_{2},\lambda_{1}^{H},\lambda_{2}^{H})=:\bar{\lambda }.$$

We shall address the following problems. Assume, that λ_1_ > λ_2_ > 0.

**Problem I**. *Are there* $0\leq \lambda_{1}^{H}\leq \lambda_{2}^{H}$ *such that L*_1_(*t*) ≤ *L*_2_(*t*) *for all large values of t?* This is investigated in Investigation 1.

**Problem II**. *Are there* $0\leq \lambda_{1}^{H}\leq \lambda_{2}^{H}$ *such that H*_1_(*t*) ≤ *H*_2_(*t*) *for all large values of t?* This is investigated in Investigation 2.

In other words we are looking for the conditions for the parameters of the system which allow the biased competition. Deco and Rolls (2005) found numerically an area of parameters which yields such effect. Here we derive this analytically. This allows us to treat a wide range of parameters and moreover to find out the exact relations between all the parameters of the network when the biased competition takes place.

## 4. Simulations of the Operation of the Network With Parameters Selected Based on the Analysis That Follows

### 4.1. Investigation 1

The top-down biased competition effect to be considered here is as follows. If λ_1_ is greater than λ_2_, then the activity of *L*_1_ will be greater than the activity of *L*_2_. But if we apply a top-down biased competition input λ_*H*_ to *H*_2_, then at some value of λ_*H*_ the top-down bias will result in *L*_2_ having as much activity as *L*_1_. The aim was to discover the critical value of λ_*H*_ by simulation, for comparison with the analytic value.

The system specified by (3.1) and (3.2) was implemented in Matlab. The parameters shown in (3.1) and (3.2) were set as follows:

*J*_*f*_ = 0.15 / 3 (the values for these synaptic weights are in the same ratio as in Deco and Rolls, 2005)

*J*_*b*_ = 0.05 / 3

*K*_*f*_ = 0.015 /3

*K*_*b*_ = 0.005 / 3

β_*L*_ = 0.35 (the decay term for the *L* nodes)

*c*_*L*_ = 0.3 (the competition between the *L* nodes)

*T*_*L*_ = 5.0 (the threshold at which an *L* node enters its attractor dynamics; in practice this was set to infinity in most of the work described, in order to prevent attractor dynamics operation in the nodes)

α_*L*_ = 0.0 (the gain factor for self-excitation in an *L* node for attractor dynamics, α_*L*_ can be set as well to 0.1 if recurrent dynamics are required)

β_*H*_ = 0.35 (the decay term for the *H* nodes)

*c*_*H*_ = 0.3 (the competition between the *H* nodes)

*T*_*H*_ = 5.0 (the threshold at which an *H* node enters its attractor dynamics; in practice this was set to infinity in most of the work described, in order to prevent attractor dynamics operation in the nodes)

α_*H*_ = 0.0 (the gain factor for self-excitation in an *H* node for attractor dynamics, α_*H*_ can be set to 0.1)

λ_1_ = 6.0

λ_2_ = 5.0

$\lambda_{2}^{H}$ = 0.0 or the critical value $\lambda_{2}^{H,cr}$ of $\lambda_{2}^{H}$ derived below in the analysis for the top-down bias to overcome the bottom-up inputs shown as λ_1_ and λ_2_.

For all of the simulations illustrated in this paper and for the analytic investigations, the recurrent collateral self-excitatory effects were turned off (i.e., the α values were set to zero, and the parameters *T*_*L*_ and *T*_*H*_ were set to infinity). However, as stated in the Legend to Figure 1, the effects obtained when these were enabled were generically the same with respect to the biased competition effects described in this research.

**Figure 2:**
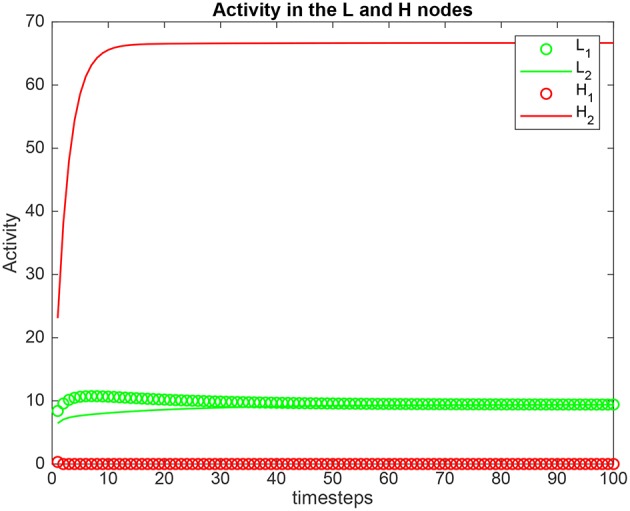
Simulation results for Investigation 1 for a system with λ_1_ = 6.0, λ_2_ = 5.0, and the top-down bias $\lambda_{2}^{H}$ = 22.816. $\lambda_{2}^{H}$ = 22.816 is the value derived in the analysis for the top-down bias to just overcome the difference between the bottom-up inputs shown as λ_1_ and λ_2_.

Simulation results for a system with λ_1_ = 6.0, λ_2_ = 5.0, and the top-down bias λ_*H*_ = $\lambda_{2}^{H,cr}$ are shown in Figure 2. $\lambda_{2}^{H,cr}$ is the value derived in the analysis for the top-down bias to just overcome the bottom-up inputs shown as λ_1_ and λ_2_, as shown below in (5.54). $\lambda_{2}^{H,cr}$ was 22.816. Now the *H*_2_ node has high activity as expected due to the application of $\lambda_{2}^{H}$, but this has the effect that the network settles into a state where the *L*_2_ node has just higher activity than the *L*_1_ node, despite λ_1_ > λ_2_. This demonstrates that the analysis described below that derives the critical value $\lambda_{2}^{H,cr}$ for the biased attention signal to just reverse the difference between the bottom up inputs to make the network respond preferentially to the weaker incoming signal, is accurate.

In further investigations, it was found that the analysis made correct predictions over a wide range of values of the parameters that were confirmed by numerical simulations. For example, the value $\lambda_{2}^{H,cr}$ was correctly calculated by the analysis over a 10-fold variation of *K*_*f*_ and *K*_*b*_ as shown by the performance of the numerical simulations. It was also found in the simulations that ratios for *J*_*b*_/*J*_*f*_ and *k*_*b*_/*k*_*f*_ in the range of 0.1–0.5 produced top-down biased competition effects with values for $\lambda_{2}^{H,cr}$ that were in the range of the other activities in the system.

**Figure 3:**
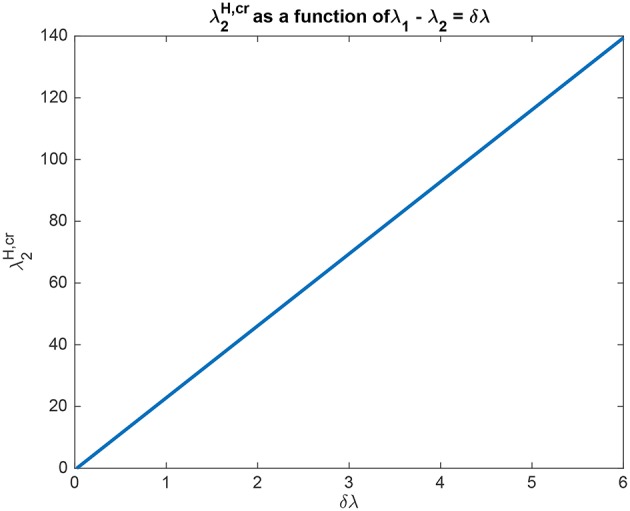
Analytical results for Investigation 1 showing the top-down bias $\lambda_{2}^{H,cr}$ needed to make the activity of *L*_2_ as large as *L*_1_ as a function of λ_1_ − λ_2_ denoted δλ. λ_1_ was fixed at 6.

The interpretation of the analytical results shown in for example (5.54) is now facilitated by graphical analysis. Figure 3 for Investigation 1 shows the top-down bias $\lambda_{2}^{H,cr}$ needed to make the activity of *L*_2_ as large as *L*_1_ as a function of λ_1_ − λ_2_ which is termed δλ. λ_1_ was fixed at 6, and $\lambda_{2}^{H}$ = 0. Figure 3 shows that $\lambda_{2}^{H,cr}$ is a linear function of δλ. The slope of this function is high (140/6). The implication is that for reasonable values of the top-down bias limited to perhaps 50 in this model, the working range for δλ is relatively small, approximately 2.

**Figure 4:**
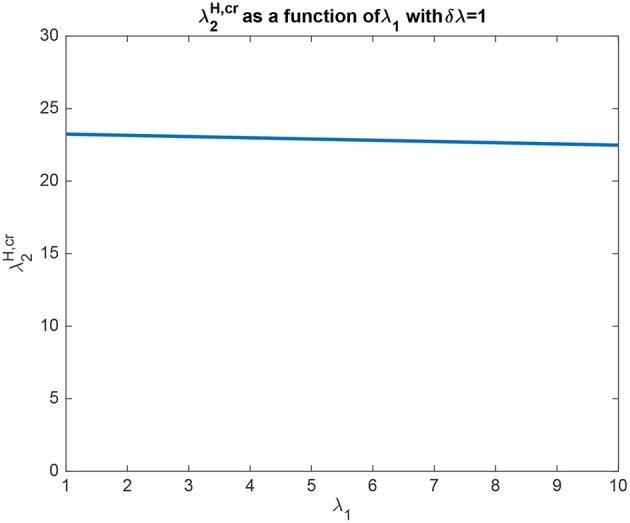
Analytical results for Investigation 1 for a system with λ_1_ − λ_2_ which is termed δλ fixed at 1. It is shown that $\lambda_{2}^{H,cr}$ the top-down bias needed to make the activity of *L*_2_ as large as *L*_1_ was relatively independent of the absolute values of λ_1_ and λ_2_.

In another example to illustrate the utility of the analysis that produced (5.54), it was found that the top-down bias $\lambda_{2}^{H,cr}$ needed to make the activity of *L*_2_ as large as *L*_1_ was relatively independent of the absolute values of λ_1_ and λ_2_, and so depended on the difference between them, i.e., λ_1_ − λ_2_ termed δλ. This is illustrated in Figure 4 in which δλ was set to 1.0, and the value of λ_1_ was increased from 1 to 10. It is clear that the top-down bias required depends very little on the absolute values of λ_1_ and λ_2_, but instead on their difference as shown in Figure 3.

We can also understand quantitatively the effect of the other parameters using (5.54). For example, if *J*_*b*_ is increased from its default value of 0.05/3 to 0.1/3, then the slope of the function illustrated in Figure 3 falls to a lower value (66/6). This makes the important point that top-down biased competition can only operate with rates for the top-down bias (in this case $\lambda_{2}^{H}$) in a reasonable range (not too high) if the backward connection strength (here *J*_*b*_ with a default value of 0.05/3) is not too low compared to the forward connection strength (here *J*_*f*_ with a default value of 0.13/3). This places important constraints on the ratio of the strengths of backprojections relative to forward projections between cortical areas (Rolls, 2016).

In another example to illustrate the utility of the analysis that produced (5.54), it was found that if the crossed connections *K*_*b*_ (default 0.005/3) were increased (for example to 0.01/3), then more top-down bias was needed, with the slope of the function shown in Figure 3 now 158/6. The reason for this is clear, that some of the top-down bias $\lambda_{2}^{H}$ acts on λ_1_ via *K*_*b*_, but the analytic result in (5.54) quantifies this, as it does the effects of the other parameters involved in this approach to biased competition.

### 4.2. Investigation 2

The second top-down competition effect considered was as follows. If λ_1_ is greater than λ_2_ and both are applied simultaneously, then the activity of *H*_1_ will be greater than the activity of *H*_2_. However, if we apply top down biased competition λ_*H*_ to *H*_2_, then we can influence the rates *H*_2_ and *H*_1_ (through all the connections in the system) until *H*_2_ and *H*_1_ have the same activity (and at the same time there will be an effect on *L*_1_ and *L*_2_). We wished to quantify these effects analytically in terms of all the parameters of the system.

**Figure 5:**
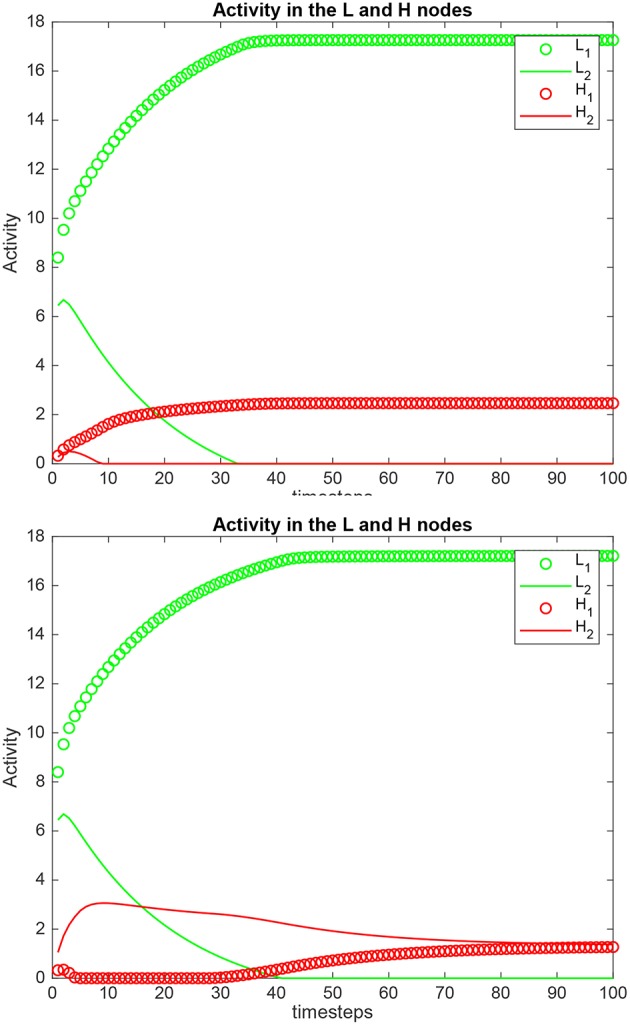
Simulation results for Investigation 2 for a system with λ_1_ = 6.0, λ_2_ = 5.0. **(Upper)** The top-down bias $\lambda_{2}^{H}$ = 0. **(Lower)** The top-down bias $\lambda_{2}^{H}$ = $\lambda_{2}^{H,cr}$ = 0.775. $\lambda_{2}^{H}$ = $\lambda_{2}^{H,cr}$ is the value derived in the analysis for the top-down bias to just produce equal rates in *H*_1_ and *H*_2_, overcoming the difference between the bottom-up inputs shown as λ_1_ and λ_2_.

Simulation results for a system with λ_1_ = 6.0, λ_2_ = 5.0, and the top-down bias $\lambda_{2}^{H}$ = 0 or = $\lambda_{2}^{H,cr}$ are shown in Figure 5. $\lambda_{2}^{H,cr}$ is the value derived in the analysis for the top-down bias to make the rates in *H*_1_ equal those in *H*_2_, to just overcome the difference between the bottom-up inputs λ_1_ and λ_2_, as shown in (5.60). The upper graph in Figure 5 shows the results when the top-down bias λ_*H*_ = 0. It can be seen that the rates in *H*_1_ are higher than in *H*_2_, which is as expected, because λ_1_ = 6.0, and λ_2_ = 5.0. In the lower part of Figure 5 the top-down bias $\lambda_{2}^{H}$ = 0.775, the value derived in the analysis for the top-down bias to just produce equal rates in *H*_1_ and *H*_2_, overcoming the difference between the bottom-up inputs shown as λ_1_ and λ_2_. The simulation illustrated in the lower part of the figure thus shows that when the analytically calculated value for $\lambda_{2}^{H,cr}$ is used, the numerical simulation confirms that this is the correct value. When a different value is used, as shown in the top part of Figure 5, then the correct results are not obtained.

**Figure 6:**
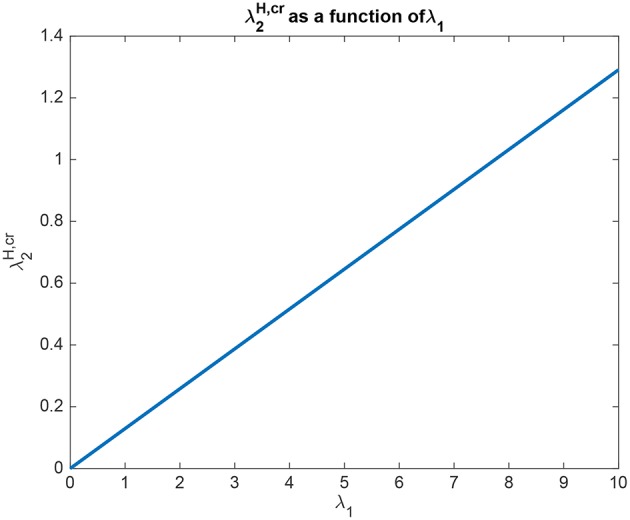
Analytical results for Investigation 2 for a system with λ_2_ = 5.0. $\lambda_{2}^{H,cr}$ is shown as a function of λ_1_. $\lambda_{2}^{H,cr}$ is the value derived in the analysis for the top-down bias to just produce equal rates in *H*_1_ and *H*_2_, overcoming the difference between the bottom-up inputs shown as λ_1_ and λ_2_.

The interpretation of the analytical results shown in for example (5.60) for Investigation 2 is now facilitated by graphical analysis. Analytical results for Investigation 2 to show how $\lambda_{2}^{H,cr}$ is a function of λ_1_ are shown in Figure 6, for a system with λ_2_ = 5.0. $\lambda_{2}^{H,cr}$ is the value derived in the analysis for the top-down bias to just produce equal rates in *H*_1_ and *H*_2_, overcoming the difference between the bottom-up inputs shown as λ_1_ and λ_2_. Figure 6 shows that $\lambda_{2}^{H,cr}$ is a linear function of λ_1_, when λ_2_ = 5.0. Moreover, Figure 6 shows that only a small variation of $\lambda_{2}^{H,cr}$ is sufficient to counteract large changes in λ_1_. Moreover, the implication of 5.60 is that provided that the conditions shown in (5.59) are met, the operation is relatively independent of λ_2_. The understanding to which this leads is that the relative outputs measured at the *H* nodes are relatively little affected by the values of λ_1_ and λ_2_, compared to the effects of the input biases to $\lambda_{2}^{H}$ and $\lambda_{1}^{H}$. An implication for the operation of the brain is that top-down biased competition can have useful effects on the lower (*L*) nodes in the system, which could then influence other systems. Another implication is that the output from the higher (*H*) nodes is relatively strongly affected by any direct inputs to the *H* nodes, compared to effects mediated by top-down biases acting through the backward connections to the *L* nodes, and on systems connected to these *L* nodes.

**Figure 7:**
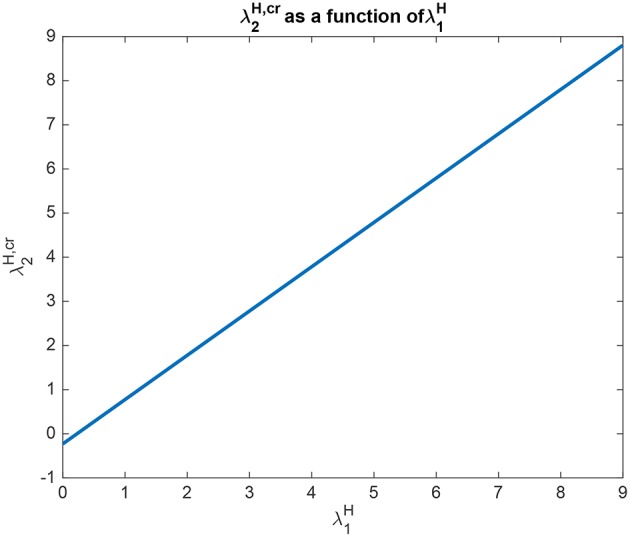
Analytical results for Investigation 2 for a system with λ_1_ = 6.0, λ_2_ = 5.0. $\lambda_{2}^{H,cr}$ is shown as a function of $\lambda_{1}^{H}$. $\lambda_{2}^{H,cr}$ is the value derived in the analysis for the top-down bias to just produce equal rates in *H*_1_ and *H*_2_, overcoming the difference between the bottom-up inputs shown as λ_1_ and λ_2_.

Analytical results for Investigation 2 to show how $\lambda_{2}^{H,cr}$ depends on $\lambda_{1}^{H}$ are shown in Figure 7, for a system with λ_2_ = 5.0. $\lambda_{2}^{H,cr}$ is the value derived in the analysis for the top-down bias to just produce equal rates in *H*_1_ and *H*_2_, overcoming the difference between the bottom-up inputs shown as λ_1_ and λ_2_. This figure shows that $\lambda_{2}^{H,cr}$ is a linear function of $\lambda_{1}^{H}$ with a slope of approximately 1. The implication here is that inputs to the *H* nodes influence each other almost equally, and this will occur primarily through the inhibition between these nodes implemented by *c*_*H*_, rather than through the top-down connections to the *L* nodes, and then the return effects from the *L* to the *H* nodes.

## 5. Mathematical Analysis

To solve the named problems we shall study the system (3.1) and (3.2) which describes the dynamics of (*L*_1_(*t*), *L*_2_(*t*), *H*_1_(*t*), *H*_2_(*t*)) in $R_{+}^{4}$. Note that the entire state space $R_{+}^{4}=\left\{\left(x_{1},x_{2},y_{1},y_{2}\right):x_{i}\geq 0,y_{i}\geq 0\right\}$ is decomposed into 2^4^ = 16 areas depending on whether *x*_*i*_ ∈ [0, *T*_*L*_], *y*_*i*_ ∈ [0, *T*_*H*_]:

(5.7) $$\begin{array}{l} R_{+}^{4}=\cup_{\left(e_{1},\ldots ,e_{4}\right):e_{i}\in \left\{0,1\right\}}X_{e_{1}}\times X_{e_{2}}\times Y_{e_{3}}\times Y_{e_{4}}, \end{array}$$

where

$$\begin{array}{l} X_{0}=\left[0,T_{L}\right),X_{1}=\left[T_{L},\infty \right),Y_{0}=\left[0,T_{H}\right),Y_{1}=\left[T_{H},\infty \right). \end{array}$$

In each area *X*_*e*_1__ × *X*_*e*_2__ × *Y*_*e*_3__ × *Y*_*e*_4__ the behavior of a system (3.1), (3.2) is linear; the non-linear nature of this system is seen, roughly speaking, only at the borders of these areas. In particular, there is a “cut” or “threshold” at zero for all involved functions, as their meaning as rates assumes only non-negative values.

We are mostly interested in modeling dynamics of a vector (*L*_1_(*t*), *L*_2_(*t*), *H*_1_(*t*), *H*_2_(*t*)) which does not escape to infinity in any coordinate. Therefore, we shall first study the system in the area

(5.8) $$\begin{array}{l} S:=\left\{\left(x_{1},x_{2},y_{1},y_{2}\right):x_{i}\in \left[0,T_{L}\right],\;y_{i}\in \left[0,T_{H}\right],\;i=1,2\right\}\\ \;=X_{0}\times X_{0}\times Y_{0}\times Y_{0}. \end{array}$$

Let us assume first the following simplifying assumption. Set

(5.9) $$\begin{array}{l} T_{L}=T_{H}=\infty , \end{array}$$

which means that there is no facilitation of rates in the network.

Consider the linear system associated with the system (3.1), (3.2):

(5.10) $$\begin{array}{l} \left(\begin{array}{c} {\tilde{L}}_{1}(t+1)\\ {\tilde{L}}_{2}(t+1)\\ {\tilde{H}}_{1}(t+1)\\ {\tilde{H}}_{2}(t+1) \end{array}\right)=\left(\begin{array}{llll} 1-\beta_{L} & -c_{L} & J_{b} & K_{b}\\ -c_{L} & 1-\beta_{L} & K_{b} & J_{b}\\ J_{f} & K_{f} & 1-\beta_{H} & -c_{H}\\ K_{f} & J_{f} & -c_{H} & 1-\beta_{H} \end{array}\right)\left(\begin{array}{c} {\tilde{L}}_{1}(t)\\ {\tilde{L}}_{2}(t)\\ {\tilde{H}}_{1}(t)\\ {\tilde{H}}_{2}(t) \end{array}\right)\\ \;+\left(\begin{array}{c} \lambda_{1}\\ \lambda_{2}\\ \lambda_{1}^{H}\\ \lambda_{2}^{H} \end{array}\right). \end{array}$$

Let us fix

(5.11) $$\bar{\lambda }\in S.$$

Then assuming also as in (3.5) the zero initial conditions

$$\begin{array}{l} \left({\tilde{L}}_{1}\left(0\right),{\tilde{L}}_{2}\left(0\right),{\tilde{H}}_{1}\left(0\right),{\tilde{H}}_{2}\left(0\right)\right)=\left(0,0,0,0\right)=\left(L_{1}\left(0\right),L_{2}\left(0\right),H_{1}\left(0\right),H_{2}\left(0\right)\right), \end{array}$$

we observe that as long as

(5.12) $$\begin{array}{l} {\tilde{L}}_{i}\left(t\right)\in \left[0,T_{L}\right],\;{\tilde{H}}_{i}\left(t\right)\in \left[0,T_{H}\right],\;i=1,2, \end{array}$$

i.e., ${\tilde{L}}_{i}\left(t\right)\geq 0$ and ${\tilde{H}}_{i}\left(t\right)\geq 0$ [recall assumption (5.9)], the system (5.10) describes exactly the same system as in (3.1) and (3.2), i.e.,

(5.13) $$\begin{array}{l} {\tilde{L}}_{i}\left(t\right)=L_{i}\left(t\right),\;{\tilde{H}}_{i}\left(t\right)=H_{i}\left(t\right). \end{array}$$

Therefore we first derive the conditions for the matrix in (5.10) under which relations (5.12) hold for all *t* ≥ 0, i.e., the system remains to be in the bounded area *S*.

The boundedness of the solution to (5.10) is defined entirely by the eigenvalues of the corresponding matrix. However, deriving the eigenvalues even for a (4 × 4)-matrix requires already heavy computations. Instead we shall take advantage of some particular properties and symmetries of our model which allow us to reduce the original 4 dimensions of the system.

### 5.1. Boundedness of the Trajectories

Let us consider the dynamics of two systems related to (5.10):

(5.14) $$L(t)={\tilde{L}}_{1}(t)+{\tilde{L}}_{2}(t),\;H(t)={\tilde{H}}_{1}(t)+{\tilde{H}}_{2}(t),$$

and

(5.15) $$\begin{array}{l} \Delta L\left(t\right)={\tilde{L}}_{1}\left(t\right)-{\tilde{L}}_{2}\left(t\right),\;\Delta H\left(t\right)={\tilde{H}}_{1}\left(t\right)-{\tilde{H}}_{2}\left(t\right). \end{array}$$

Observe that **H**(*t*) → ∞ or Δ*H*(*t*) → ∞ if and only if at least one ${\tilde{H}}_{i}\left(t\right)\to \infty$, in which case the original system (*L*_1_(*t*), *L*_2_(*t*), *H*_1_(*t*), *H*_2_(*t*)) cannot remain in the bounded area *S*. A similar statement holds about **L**(*t*) and Δ*L*(*t*). Therefore, we begin the analysis by providing the necessary conditions for the stability of systems (5.14) and (5.15), and thus for the stability of (5.10).

Then assuming that the connection parameters provide stability for the sums (5.14) we investigate the behavior of the system of differences (5.15) for different inputs

$$\lambda_{1}>\lambda_{2}\;\text{and }\lambda_{1}^{H}<\lambda_{2}^{H}.$$

Our goal here is to find parameters such that given asymmetric inputs λ_1_ > λ_2_ we want to find $\lambda_{1}^{H}<\lambda_{2}^{H}$ such that Δ*L*(*t*) converges to a negative value for large *t* or that Δ*H*(*t*) converges to a negative value for large *t*.

If the equality (5.13) still holds for all *t* this means that correspondingly, *L*_1_(*t*) < *L*_2_(*t*) or *H*_1_(*t*) < *H*_2_(*t*) for large *t* as well. Hence, correspondingly, we have a solution to Problem I or Problem II.

First we derive from (5.10):

(5.16) $$\left(\begin{array}{c} L(t+1)\\ H(t+1) \end{array}\right)=W_{S}\left(\begin{array}{c} L(t)\\ H(t) \end{array}\right)+\left(\begin{array}{c} \lambda \\ \lambda^{H} \end{array}\right),$$

where

(5.17) $$\begin{array}{l} W_{S}=\left(\begin{array}{l} 1-\beta_{L}-c_{L}J_{b}+K_{b}\\ J_{f}+K_{f}1-\beta_{H}-c_{H} \end{array}\right), \end{array}$$

and

$$\begin{array}{l} \lambda =\lambda_{1}+\lambda_{2},\;\lambda^{H}=\lambda_{1}^{H}+\lambda_{2}^{H}. \end{array}$$

Similarly,

(5.18) $$\begin{array}{l} \left(\begin{array}{c} \Delta L\left(t+1\right)\\ \Delta H\left(t+1\right) \end{array}\right)=W_{\Delta }\left(\begin{array}{c} \Delta L\left(t\right)\\ \Delta H\left(t\right) \end{array}\right)+\left(\begin{array}{c} \Delta \lambda \\ \Delta \lambda^{H} \end{array}\right), \end{array}$$

where

(5.19) $$\begin{array}{l} W_{\Delta }=\left(\begin{array}{l} 1-\beta_{L}+c_{L}J_{b}-K_{b}\\ J_{f}-K_{f}1-\beta_{H}+c_{H} \end{array}\right), \end{array}$$

and

$$\begin{array}{l} \Delta \lambda =\lambda_{1}-\lambda_{2},\;\Delta \lambda^{H}=\lambda_{1}^{H}-\lambda_{2}^{H}. \end{array}$$

Effectively, using the symmetries we reduced the 4 dimensions of our model to 2 dimensions, as the solution to our original system (5.10) is given by

(5.20) $${\tilde{L}}_{1}(t)=\frac{1}{2}(L(t)+\Delta L(t)),\;{\tilde{L}}_{2}(t)=\frac{1}{2}(L(t)-\Delta L(t)),$$

and

(5.21) $${\tilde{H}}_{1}(t)=\frac{1}{2}(H(t)+\Delta H(t)),\;{\tilde{H}}_{2}(t)=\frac{1}{2}(H(t)-\Delta H(t)).$$

Recall that for a 2x2 matrix

(5.22) $$\begin{array}{l} M=\left(\begin{array}{c} AC\\ DB \end{array}\right), \end{array}$$

where all the entries are positive (as in our model) the eigenvalues are given by

(5.23) $$\begin{array}{l} \kappa_{1}=\frac{A+B+\sqrt{{\left(A-B\right)}^{2}+4CD}}{2}, \end{array}$$

and

(5.24) $$\begin{array}{l} \kappa_{2}=\frac{A+B-\sqrt{{\left(A-B\right)}^{2}+4CD}}{2}. \end{array}$$

Note that due to the assumption of positivity of entries we have here

(5.25) $$\begin{array}{l} \kappa_{2}<\kappa_{1},\;|\kappa_{2}|<\kappa_{1}. \end{array}$$

Hence, we have two (not linearly dependent) eigenvectors

(5.26) $$\begin{array}{l} E_{i}=\left(\begin{array}{c} 1\\ \frac{\kappa_{i}-A}{C} \end{array}\right). \end{array}$$

Note here for further reference that the inequalities

(5.27) $$\begin{array}{l} \kappa_{1}-A=\frac{B-A+\sqrt{{\left(A-B\right)}^{2}+4CD}}{2}>0, \end{array}$$

and

(5.28) $$\begin{array}{l} \kappa_{2}-A=\frac{B-A-\sqrt{{\left(A-B\right)}^{2}+4CD}}{2}<0 \end{array}$$

hold for all positive parameters *A, B, C, D*.

Let us consider a linear transformation in *R*^2^:

(5.29) $$\begin{array}{l} \overset{-}{X}\;\left(t+1\right)=M\overset{-}{X}\;\left(t\right)+ȳ, \end{array}$$

where $\overset{-}{X}\left(0\right)=\left(0,0\right)$ and ȳ = (*y*_1_, *y*_2_) is any vector.

Since vectors *E*_1_ and *E*_2_ make a basis in *R*^2^, for any vector ȳ there are numbers *q*_1_ and *q*_2_, such that

(5.30) $$\begin{array}{l} ȳ=q_{1}E_{1}+q_{2}E_{2}=\left(\begin{array}{c} q_{1}+q_{2}\\ \frac{\kappa_{1}-A}{C}q_{1}+\frac{\kappa_{2}-A}{C}q_{2}. \end{array}\right). \end{array}$$

Then the solution to (5.29) is given by

(5.31) $$\begin{array}{l} \overset{-}{X}\left(t+1\right)=\overset{t}{\underset{k=0}{\sum }}M^{k}ȳ=\overset{t}{\underset{k=0}{\sum }}\kappa_{1}^{k}q_{1}E_{1}+\overset{t}{\underset{k=0}{\sum }}\kappa_{2}^{k}q_{2}E_{2}. \end{array}$$

Observe that

(5.32) $$\kappa_{1}<1\text{ if and only if }\{\begin{array}{l} A+B<2,\\ CD<(1-A)(1-B). \end{array}$$

The last system together with the assumption that both *C* and *D* are positive yields

(5.33) $$\kappa_{1}<1\text{ if and only if }\{\begin{array}{l} 0<A,B<1,\\ CD<(1-A)(1-B). \end{array}$$

Under the last condition equation (5.31) yields the following convergence to the fixed point:

(5.34) $$\begin{array}{l} \overset{-}{X}\left(t\right)\to \frac{1}{1-\kappa_{1}}q_{1}E_{1}+\frac{1}{1-\kappa_{2}}q_{2}E_{2}=\left(\begin{array}{c} \frac{q_{1}}{1-\kappa_{1}}+\frac{q_{2}}{1-\kappa_{2}}\\ \frac{\kappa_{1}-A}{C\left(1-\kappa_{1}\right)}q_{1}+\frac{\kappa_{2}-A}{C\left(1-\kappa_{2}\right)}q_{2} \end{array}\right), \end{array}$$

as *t* → ∞.

Consider *W*_*S*_ defined in (5.17) as an example of the generic matrix *M* in (5.22). Let us denote its eigenvalues κ_*i*_(*W*_*S*_), *i* = 1, 2, κ_1_(*W*_*S*_) ≥ κ_2_(*W*_*S*_). Under assumption

(5.35) $$\begin{array}{l} \beta_{L}+c_{L}<1,\;\beta_{H}+c_{H}<1, \end{array}$$

we have according to (5.33):

(5.36) $$\begin{array}{l} \kappa_{1}\left(W_{S}\right)<1\text{ if and only if}\left(J_{f}+K_{f}\right)\left(J_{b}+K_{b}\right)<\left(\beta_{L}+c_{L}\right)\left(\beta_{H}+c_{H}\right). \end{array}$$

Next we study the eigenvalues κ_1_(*W*_Δ_) ≥ κ_2_(*W*_Δ_) of *W*_Δ_. Under assumption

(5.37) $$\begin{array}{l} \beta_{L}-c_{L}<1,\;\beta_{H}-c_{H}<1, \end{array}$$

which holds whenever (5.35) holds, we have according to (5.33):

(5.38) $$\begin{array}{l} \kappa_{1}\left(W_{\Delta }\right)<1\text{ if and only if}\left(J_{f}-K_{f}\right)\left(J_{b}-K_{b}\right)<\left(\beta_{L}-c_{L}\right)\left(\beta_{H}-c_{H}\right). \end{array}$$

Observe that when *c*_*L*_ = *c*_*H*_ = 0, condition (5.38) follows by the above condition (5.36).

Notice that together with the assumption (3.3) when all the parameters except *J*_*b*_, *K*_*b*_ are fixed, the ratio *q* = *J*_*b*_/*J*_*f*_ has to be small to ensure the boundedness of the functions *L*_*i*_(*t*), *H*_*i*_(*t*).

In words, conditions (5.36) and (5.38) tell us that under the assumption (5.35) the stability of the system (or boundedness of the trajectories) requires that at least some of the connections, forward or backward ones, have to be sufficiently small.

### 5.2. Dynamics of (Δ*L*(*t*), Δ*H*(*t*)) for a Biased Input When All Rates Remain to be Strictly Positive

Here we study the system (3.1)–(3.2) assuming conditions when the functions *L*_*i*_(*t*), *H*_*i*_(*t*) remain to be strictly positive and bounded. This means that the dynamics is described by linear system.

Assume that initially the input to ${\tilde{L}}_{1}$ is greater than the one to ${\tilde{L}}_{2}$, i.e., Δλ > 0. We shall find here the sufficient conditions on the parameters of the connections which yield existence of the values

$$\begin{array}{l} \Delta \lambda^{H}<0 \end{array}$$

such that eventually, contrary to the initial bias, the state of the system satisfies

(5.39) $$\begin{array}{l} {\tilde{L}}_{2}\left(t\right)<{\tilde{L}}_{1}\left(t\right), \end{array}$$

for all large *t*.

We shall assume first of all that the above conditions (5.35), (5.36), (5.37), and (5.38) are satisfied.

Consider system (5.19) with input

$$\begin{array}{l} \left(\begin{array}{c} \Delta \lambda \\ \Delta \lambda^{H} \end{array}\right)=\left(\begin{array}{c} \lambda_{1}-\lambda_{2}\\ \lambda_{1}^{H}-\lambda_{2}^{H} \end{array}\right). \end{array}$$

Let us decompose this vector along the eigenvectors of *W*_Δ_ as in (5.30):

(5.40) $$\begin{array}{l} \left(\begin{array}{c} \Delta \lambda \\ \Delta \lambda^{H} \end{array}\right)=xE_{1}\left(W_{\Delta }\right)\;+\;yE_{2}\left(W_{\Delta }\right)=\left(\begin{array}{c} x+y\\ \frac{\kappa_{1}\left(W_{\Delta }\right)-A_{\Delta }}{C_{\Delta }}x+\frac{\kappa_{2}\left(W_{\Delta }\right)-A_{\Delta }}{C_{\Delta }}y \end{array}\right), \end{array}$$

where *E*_*i*_(*W*_Δ_) and κ_*i*_(*W*_Δ_), *i* = 1, 2, denote, correspondingly the eigenvectors and the eigenvalues of *W*_Δ_, and here [compare matrices (5.19) and (5.22)]

$$\begin{array}{l} C_{\Delta }=J_{b}-K_{b},\;A_{\Delta }=1-\beta_{L}+c_{L}. \end{array}$$

Then by (5.34) the following convergence takes place when *t* → ∞:

(5.41) $$\begin{array}{l} \left(\begin{array}{c} \Delta L\left(t\right)\\ \Delta H\left(t\right) \end{array}\right)\to \left(\begin{array}{c} \frac{x}{1-\kappa_{1}\left(W_{\Delta }\right)}+\frac{y}{1-\kappa_{2}\left(W_{\Delta }\right)}\\ \frac{\kappa_{1}\left(W_{\Delta }\right)-A_{\Delta }}{C_{\Delta }\left(1-\kappa_{1}\left(W_{\Delta }\right)\right)}x+\frac{\kappa_{2}\left(W_{\Delta }\right)-A_{\Delta }}{C_{\Delta }\left(1-\kappa_{2}\left(W_{\Delta }\right)\right)}y \end{array}\right). \end{array}$$

Hence, given a positive

$$\Delta \lambda =\lambda_{1}-\lambda_{2}=x+y$$

we want to find value *x* = *x*(Δλ) which satisfies condition

(5.42) $$\begin{array}{l} \frac{x}{1-\kappa_{1}}+\frac{\Delta \lambda -x}{1-\kappa_{2}}<0 \end{array}$$

and moreover minimizes function [see (5.40)]

(5.43) $$\begin{array}{l} F\left(x\right)=\left|\frac{\kappa_{1}-A_{\Delta }}{C_{\Delta }}x+\frac{\kappa_{2}-A_{\Delta }}{C_{\Delta }}\left(\Delta \lambda -x\right)\right|. \end{array}$$

From (5.42) we get

(5.44) $$\begin{array}{l} x<-\Delta \lambda \frac{1-\kappa_{1}}{\kappa_{1}-\kappa_{2}}, \end{array}$$

which is negative. Then for all the *x* which satisfy (5.44) we have

(5.45) $$\begin{array}{c} F\left(x\right)=\frac{1}{C_{\Delta }}|\left(\kappa_{1}-\kappa_{2}\right)x-\left(A_{\Delta }-\kappa_{2}\right)\Delta \lambda |\\ =\frac{\left(\kappa_{1}-\kappa_{2}\right)|x|+\left(A_{\Delta }-\kappa_{2}\right)\Delta \lambda }{C_{\Delta }}>\Delta \lambda \;\frac{1+A_{\Delta }-\left(\kappa_{1}+\kappa_{2}\right)}{C_{\Delta }}. \end{array}$$

Finally, we can define a negative Δλ^*H*^ such that (5.40) holds, and moreover the limiting state as defined in the first row on the right in (5.41) satisfies (5.42). Observe that condition (5.42) by (5.41) guarantees condition (5.39). Substituting previously derived formulas into (5.45) we derive that for any given positive Δλ and any $\lambda_{1}^{H}<\lambda_{2}^{H}$ such that

(5.46) $$\begin{array}{l} \lambda_{2}^{H}-\lambda_{1}^{H}>\frac{\beta_{H}-c_{H}}{J_{b}-K_{b}}\Delta \lambda \end{array}$$

we have (5.39).

We conclude that in the case when (5.13) still holds for all large *t*, i.e., if the system remains to be in the positive area, we have *L*_2_(*t*) ≥ *L*_1_(*t*) for all large *t* if conditions (5.35), (5.36), and (5.38) are fulfilled and $\lambda_{2}^{H}$ is greater than the following critical value:

(5.47) $$\begin{array}{l} \lambda_{2}^{H,cr}=\lambda_{1}^{H}+\frac{\beta_{H}-c_{H}}{J_{b}-K_{b}}\left(\lambda_{1}-\lambda_{2}\right). \end{array}$$

### 5.3. Operation With a Threshold at a Rate of Zero

Here we explore the non-linear effects of the system (3.1)–(3.2) considering the case when some of the rates *L*_*i*_(*t*) or *H*_*i*_(*t*) become zero and may stay at zero due to the non-linear threshold function that does not allow negative rates (·)^+^.

#### 5.3.1. Equality in the *L*-Nodes

We shall find here the conditions when a state

(5.48) $$\begin{array}{l} L_{1}\left(t\right)=L_{2}\left(t\right)=L,H_{1}\left(t\right)=0,H_{2}\left(t\right)=H \end{array}$$

with strictly positive *L* and *H* can be a fixed point for the dynamical system (3.1)-(3.2) under assumption that

$$\begin{array}{l} \lambda_{1}>\lambda_{2},\lambda_{2}^{H}>\lambda_{1}^{H} \end{array}$$

and also (5.9) holds, i.e., when a facilitation of rate above a certain threshold is not applied.

Assuming that the trajectories of *L*_1_(*t*), *L*_2_(*t*), and *H*_2_(*t*) remain to be strictly positive and do not hit zero, while *H*_1_(*t*) does decay to zero, the constants in (5.48) should satisfy the following system derived from (5.10) [see also 3.1)–(3.2)]:

(5.49) $$\left\{ \begin{array}{l} -\beta_{L}L-c_{L}L+K_{b}H+\lambda_{1}=0,\\ -c_{L}L-\beta_{L}L+J_{b}H+\lambda_{2}=0,\\ J_{f}L+K_{f}L-c_{H}H+\lambda_{1}^{H}\leq 0,\\ K_{f}L+J_{f}L-\beta_{H}H+\lambda_{2}^{H}=0. \end{array}\right.$$

Observe that the inequality in the third line in (5.49) after the threshold at a rate of zero as in the original system (3.1)-(3.2) yields limiting state *H*_1_(*t*) = 0.

The system (5.49) is equivalent to

(5.50) $$\left\{ \begin{array}{l} 0<H=\frac{\lambda_{1}-\lambda_{2}}{J_{b}-K_{b}}\leq \frac{\lambda_{2}^{H}-\lambda_{1}^{H}}{\beta_{H}-c_{H}},\\ L=\frac{J_{b}H+\lambda_{2}}{\beta_{L}+c_{L}}=\frac{\beta_{H}H-\lambda_{2}^{H}}{K_{f}+J_{f}}. \end{array}\right.$$

This requires the following conditions for the parameters in order for the last system to have a solution:

(5.51) $$\begin{array}{c} \frac{\lambda_{2}\left(K_{f}+J_{f}\right)+\left(\frac{\lambda_{1}-\lambda_{2}}{J_{b}-K_{b}}\left(\beta_{H}-c_{H}\right)+\lambda_{1}^{H}\right)\left(\beta_{L}+c_{L}\right)}{\beta_{H}\left(\beta_{L}+c_{L}\right)-J_{b}\left(K_{f}+J_{f}\right)}\leq H\\ =\frac{\lambda_{2}\left(K_{f}+J_{f}\right)+\lambda_{2}^{H}\left(\beta_{L}+c_{L}\right)}{\beta_{H}\left(\beta_{L}+c_{L}\right)-J_{b}\left(K_{f}+J_{f}\right)}=\frac{\lambda_{1}-\lambda_{2}}{J_{b}-K_{b}}, \end{array}$$

which in turn requires

(5.52) $$\begin{array}{l} \frac{\lambda_{2}\left(K_{f}+J_{f}\right)\left(J_{b}-K_{b}\right)+\left(\left(\lambda_{1}-\lambda_{2}\right)\left(\beta_{H}-c_{H}\right)+\lambda_{1}^{H}\left(J_{b}-K_{b}\right)\right)\left(\beta_{L}+c_{L}\right)}{\beta_{H}\left(\beta_{L}+c_{L}\right)-J_{b}\left(K_{f}+J_{f}\right)}\leq \lambda_{1}-\lambda_{2}. \end{array}$$

Observe that when $\lambda_{1}^{H}=0$ the last condition is equivalent to

(5.53) $$\begin{array}{l} \left(\lambda_{1}J_{b}-\lambda_{2}K_{b}\right)\left(K_{f}+J_{f}\right)\leq \left(\lambda_{1}-\lambda_{2}\right)c_{H}\left(\beta_{L}+c_{L}\right). \end{array}$$

Assuming that (5.52) holds we derive from (5.51) the following *critical value*

(5.54) $$\begin{array}{l} \lambda_{2}^{H,cr}=\frac{\lambda_{1}-\lambda_{2}}{J_{b}-K_{b}}\left(\beta_{H}-J_{b}\frac{K_{f}+J_{f}}{\beta_{L}+c_{L}}\right)-\lambda_{2}\frac{K_{f}+J_{f}}{\beta_{L}+c_{L}}. \end{array}$$

The above analysis yields the following statement.

**Proposition 5.1**. *Let conditions (5.36) and (5.38) hold, and let*

$$\lambda_{2}^{H}\geq \lambda_{2}^{H,cr}.$$

*Assume also (5.52) (or (5.53) if* $\lambda_{1}^{H}=0$) *and*

(5.55) $$\begin{array}{l} \frac{\lambda_{2}\left(K_{f}+J_{f}\right)+\lambda_{2}^{H}\left(\beta_{L}+c_{L}\right)}{\beta_{H}\left(\beta_{L}+c_{L}\right)-J_{b}\left(K_{f}+J_{f}\right)}=\frac{\lambda_{1}-\lambda_{2}}{J_{b}-K_{b}}. \end{array}$$

Then the system (3.1)-(3.2) converges to a state where

$$H_{1}\left(t\right)=0,\;H_{2}\left(t\right)=H=\frac{\lambda_{1}-\lambda_{2}}{J_{b}-K_{b}},\;L_{1}\left(t\right)=L_{2}\left(t\right)=\frac{J_{b}H+\lambda_{2}}{\beta_{L}+c_{L}}.$$

Notice that the limiting state described in the last Proposition satisfies (5.48).

Observe that the formula (5.54) for the critical value $\lambda_{2}^{H,cr}$ is in a good agreement with the previous case (5.47); in fact the same condition as in (5.47) reads directly from the inequality in (5.50). However, this is precisely the non-linearity of the system that we use here to derive the exact formula (5.54) for the critical value.

#### 5.3.2. Equality in the *H*-Nodes

We shall find here the conditions when a state

(5.56) $$\begin{array}{l} L_{1}\left(t\right)=L,L_{2}\left(t\right)=0,H_{1}\left(t\right)=H_{2}\left(t\right)=H \end{array}$$

with strictly positive *L* and *H* can be a fixed point for the dynamical system (3.1)-(3.2) under the assumption that

$$\begin{array}{l} \lambda_{1}>\lambda_{2},\lambda_{2}^{H}>\lambda_{1}^{H} \end{array}$$

and also (5.9) holds, i.e., when a facilitation of rate above a certain threshold is not applied.

Assuming that the trajectories of *L*_1_(*t*), *H*_1_(*t*), and *H*_2_(*t*) remain to be strictly positive and do not hit zero, while *L*_2_(*t*) does decay to zero, the constants in (5.56) should satisfy the following system derived from (5.10) [see also 3.1)–(3.2)]:

(5.57) $$\{\begin{array}{c} -\beta_{L}L+(J_{b}+K_{b})H+\lambda_{1}=0,\\ -c_{L}L+(J_{b}+K_{b})H+\lambda_{2}\leq 0,\\ J_{f}L-(\beta_{H}+c_{H})H+\lambda_{1}^{H}=0,\\ K_{f}L-(\beta_{H}+c_{H})H+\lambda_{2}^{H}=0. \end{array}$$

Observe that the inequality in the second line in (5.57) after the threshold at zero as in the original system (3.1)–(3.2) yields limiting state *L*_2_(*t*) = 0. The system (5.57) is equivalent to

(5.58) $$\left\{ \begin{array}{l} 0<L=\frac{\lambda_{2}^{H}-\lambda_{1}^{H}}{J_{f}-K_{f}}\leq \frac{\lambda_{1}-\lambda_{2}}{\beta_{L}-c_{L}},\\ H=\frac{K_{f}L+\lambda_{2}^{H}}{\beta_{H}+c_{H}}=\frac{\beta_{L}L-\lambda_{1}}{K_{b}+J_{b}}. \end{array}\right.$$

First we derive the conditions for the parameters in order for the last system to have a solution:

(5.59) $$\begin{array}{l} \lambda_{1}\left(c_{L}-J_{f}\frac{K_{b}+J_{b}}{\beta_{H}+c_{H}}\right)\geq \lambda_{1}^{H}\frac{K_{b}+J_{b}}{\beta_{H}+c_{H}}\left(\beta_{L}\;-\;c_{L}\right)\;+\;\lambda_{2}\left(\beta_{L}-J_{f}\frac{K_{b}+J_{b}}{\beta_{H}+c_{H}}\right). \end{array}$$

Assuming that the latter holds we derive from (5.57) the following critical value

(5.60) $$\begin{array}{l} \lambda_{2}^{H,cr}=\frac{\lambda_{1}\left(J_{f}-K_{f}\right)\left(\beta_{H}+c_{H}\right)+\lambda_{1}^{H}\left(\left(\beta_{H}+c_{H}\right)\beta_{L}-K_{f}\left(K_{b}+J_{b}\right)\right)}{\left(\beta_{H}+c_{H}\right)\beta_{L}-\left(J_{b}+K_{b}\right)J_{f}}. \end{array}$$

If $\lambda_{2}^{H}\geq \lambda_{2}^{H,cr}$ then the system ends up in a state where *L*_1_(*t*) = *L* > *L*_2_(*t*) = 0 and *H*_2_(*t*) = *H*_1_(*t*) = *H*.

## 6. Discussion

### 6.1. The Analysis

We consider here a 4-dimensional system of linear equations with thresholds for the rates at zero. Although it is “almost” a linear system, which admits rather straightforward analysis, the focus is on the relations between the numerous parameters. Notably, the latter relations are mostly non-linear.

Our approach takes advantage of the authentic symmetries in the system which allowed us to reduce the original 4 dimensions to 2 dimensions. This method may have some interest on its own as it can be used in other similar situations.

The derived conditions for the parameters which yield certain desired properties (specified as Problems I and II) disclose non-trivial relations between the parameters.

We considered several cases: (i) when the system keeps a positive rate at each node (section 5.2), and (ii) when the rate at one node, namely *L*_2_(*t*) (section 5.3.2), or *H*_2_(*t*) (section 5.3.1) is suppressed to zero after a long enough time.

Consider first our solution to Problem I. Remarkably the formulas for the critical value of the bias $\lambda_{2}^{H}$ to yield the success of competition are different for the above two cases, namely when all the rates are strictly positive or when one rate is zero. These formulas are given by (5.47) and (5.54). This level of accuracy would not be possible without analytic formulas. Observe that (5.47) is the first condition in system (5.50), which also has to be fulfilled for the formula (5.54) to work.

As we mentioned above, formula (5.47) holds only under assumption that all the rates remain to be strictly positive, that can be achieved, for example, by choosing $\lambda_{1}^{H}$ sufficiently large.

Further, we notice that the solution to Problem II provided by formula (5.60) reveals an interesting relation: as long as λ_2_ satisfies condition (5.59) it does not enter directly formula (5.60).

Deco and Rolls (2005) inferred from their particular simulations that the ratio of 2.5 between *J*_*f*_ and *J*_*b*_ provides a good working point for the biased competition. As we do not find any universal ratio between *J*_*f*_ and *J*_*b*_ in our analysis we conclude that the ratio 2.5 reflects particular scaling when the remaining parameters are fixed at certain values. On the other hand, our analysis tells us that the product *J*_*f*_*J*_*b*_ has to be sufficiently small for the boundedness of the trajectories. More precisely, (5.36) and (5.38) under assumption (3.3) require the following sufficient conditions for our analysis

(6.61) $$\begin{array}{l} q=\frac{J_{b}}{J_{f}}<\text{ min }\left\{\frac{\left(\beta_{L}+c_{L}\right)\left(\beta_{H}+c_{H}\right)}{{\left(J_{f}+K_{f}\right)}^{2}},\;\frac{\left(\beta_{L}-c_{L}\right)\left(\beta_{H}-c_{H}\right)}{{\left(J_{f}-K_{f}\right)}^{2}}\right\} \end{array}$$

Furthermore, each of formulas (5.54) or (5.47) works under additional conditions as specified in the text. In particular, formula (5.54) requires (5.53), which is

(6.62) $$\begin{array}{l} q=\frac{J_{b}}{J_{f}}<c_{H}\;\frac{\lambda_{1}-\lambda_{2}}{\lambda_{1}J_{f}-\lambda_{2}K_{f}}\frac{\beta_{L}+c_{L}}{K_{f}+J_{f}}. \end{array}$$

A reasonably large set of parameters satisfies the above conditions, as shown by the computational results.

### 6.2. Implications for Understanding Biased Competition and the Interaction Between Neural Systems

The analysis elucidates some interesting properties of the biased competition system described. For example, the system is sensitive to the difference between λ_1_ and λ_2_, with the amount of biased competition required to produce the biased competition effects described related to this difference, as shown by the analytical results leading to (5.54), and the results shown in Figures 3, 4. These analyses and results show that while the system tolerates a wide range for the absolute values of λ_1_ and λ_2_, the difference between then δλ must be relatively small for the values of the top-down bias $\lambda_{2}^{H,cr}$ to be within a reasonable range of activity values, which in the context of the simulations described here might be up to 50.

The mean value of the λ inputs on the other hand influences how high the rates are of the output neurons. Another feature revealed by the analysis is how the parameters can be set to achieve asymptotically stable performance.

The analysis has interesting implications for understanding the operation of the backprojections that are important in top-down biased competition mechanisms of attention. Equation (5.54) and Figure 3, the associated results show that in order for the top down critical value $\lambda_{2}^{H,cr}$ not to have to be too large, the backprojections *J*_*b*_ must not be too weak. At the same time, *J*_*b*_ must be less than *J*_*f*_, so that perceptual bottom-up inputs can dominate neural processing, which must not be dominated by internally generated top-down signals. This leaves a relatively small region for *J*_*b*_/*J*_*f*_ between perhaps < 1.0 and 0.3. However, this ratio must be kept fairly low so that the two systems being coupled in this way can operate with separate attractors at the bottom (*L*) and top (*H*) ends (Renart et al., 1999a,b).

Another interesting property of this top-down biased competition system elucidated by the analysis is that the operation of the system, including the effects of the top-down bias $\lambda_{2}^{H}$, was influenced especially by the difference δλ between λ_1_ and λ_2_, rather than by their absolute value, as shown in (5.54) and illustrated in Figures 3, 4. This is similar to the operation of integrate-and-fire decision-making networks (Rolls and Deco, 2010; Deco et al., 2013; Rolls, 2016), with the similarity reflecting the way in which the competition between the nodes or attractors operates.

The key correspondence between the mathematical analysis and the numerical simulations is that the simulations show that the mathematical analysis very accurately specifies the exact value of the top-down bias that is needed. That is useful confirmation that the analysis accurately specifies the interactions between the parameters in the biased competition system. The simulations are additionally useful in illustrating the operation of the biased competition system investigated analytically.

In conclusion, the major advantage of the analytical approach brought to bear here for the first time on biased competition between cortical areas is that it discloses relations between all the parameters of the model, and helps to identify those values that yield the desired effect of biased competition. This task cannot be fulfilled purely by numerical simulations.

## Data Availability

All datasets analyzed for this study are included in the manuscript and the supplementary files.

## Author Contributions

TT performed the mathematical analyses. ER performed the numerical simulations and assessments. ER and TT wrote the paper jointly.

### Conflict of Interest Statement

The authors declare that the research was conducted in the absence of any commercial or financial relationships that could be construed as a potential conflict of interest.

## References

[B1] Battaglia F. Treves A. (1998). Stable and rapid recurrent processing in realistic autoassociative memories. Neural Comput. 10, 431–450. 10.1162/089976698300017827 9472489

[B2] Chelazzi L. (1998). Serial attention mechanisms in visual search: a critical look at the evidence. Psychol. Res. 62, 195–219. 10.1007/s004260050051 10472200

[B3] Chelazzi L. Miller E. Duncan J. Desimone R. (1993). A neural basis for visual search in inferior temporal cortex. Nature 363, 345–347. 10.1038/363345a0 8497317

[B4] Corchs S. Deco G. (2002). Large-scale neural model for visual attention: integration of experimental single cell and fMRI data. Cereb. Cortex 12, 339–348. 10.1093/cercor/12.4.339 11884349

[B5] Corchs S. Deco G. (2004). Feature-based attention in human visual cortex: simulation of fMRI data. Neuroimage 21, 36–45. 10.1016/j.neuroimage.2003.08.045 14741640

[B6] Deco G. Lee T. S. (2002). A unified model of spatial and object attention based on inter-cortical biased competition. Neurocomputing 44–46, 775–781. 10.1016/S0925-2312(02)00471-X

[B7] Deco G. Pollatos O. Zihl J. (2002). The time course of selective visual attention: theory and experiments. Vision Res. 42, 2925–2945. 10.1016/S0042-6989(02)00358-9 12450503

[B8] Deco G. Rolls E. T. (2002). Object-based visual neglect: a computational hypothesis. Eur. J. Neurosci. 16, 1994–2000. 10.1046/j.1460-9568.2002.02279.x 12453063

[B9] Deco G. Rolls E. T. (2003). Attention and working memory: a dynamical model of neuronal activity in the prefrontal cortex. Eur. J. Neurosci. 18, 2374–2390. 10.1046/j.1460-9568.2003.02956.x 14622200

[B10] Deco G. Rolls E. T. (2004). A neurodynamical cortical model of visual attention and invariant object recognition. Vision Res. 44, 621–644. 10.1016/j.visres.2003.09.037 14693189

[B11] Deco G. Rolls E. T. (2005). Neurodynamics of biased competition and cooperation for attention: a model with spiking neurons. J. Neurophysiol. 94, 295–313. 10.1152/jn.01095.2004 15703227

[B12] Deco G. Rolls E. T. Albantakis L. Romo R. (2013). Brain mechanisms for perceptual and reward-related decision-making. Prog. Neurobiol. 103, 194–213. 10.1016/j.pneurobio.2012.01.010 22326926

[B13] Deco G. Rolls E. T. Horwitz B. (2004). ‘What’ and ‘where’ in visual working memory: a computational neurodynamical perspective for integrating fMRI and single-neuron data. J. Cogn. Neurosci. 16, 683–701. 10.1162/089892904323057380 15165356

[B14] Deco G. Zihl J. (2001). Top-down selective visual attention: a neurodynamical approach. Vis. Cogn. 8, 119–140. 10.1080/13506280042000054

[B15] Desimone R. Duncan J. (1995). Neural mechanisms of selective visual attention. Annu. Rev. Neurosci. 18, 193–222. 10.1146/annurev.ne.18.030195.001205 7605061

[B16] Duncan J. (1996). Cooperating brain systems in selective perception and action, in Attention and Performance XVI, eds Inui T. McClelland J. L. (Cambridge, MA: MIT Press), 549–578.

[B17] Duncan J. Humphreys G. (1989). Visual search and stimulus similarity. Psychol. Rev. 96, 433–458. 10.1037//0033-295X.96.3.433 2756067

[B18] Elston G. N. (2002). Cortical heterogeneity: implications for visual processing and polysensory integration. J. Neurocytol. 31, 317–335. 10.1023/A:1024182228103 12815250

[B19] Elston G. N. Fujita I. (2014). Pyramidal cell development: postnatal spinogenesis, dendritic growth, axon growth, and electrophysiology. Front. Neuroanat. 8:78. 10.3389/fnana.2014.00078 25161611 PMC4130200

[B20] Grossberg S. (1988). Non-linear neural networks: principles, mechanisms, and architectures. Neural Netw. 1, 17–61. 10.1016/0893-6080(88)90021-4

[B21] Heinke D. Deco G. Zihl J. Humphreys G. (2002). A computational neuroscience account of visual neglect. Neurocomputing 44–46, 811–816. 10.1016/S0925-2312(02)00477-0

[B22] Kastner S. De Weerd P. Desimone R. Ungerleider L. (1998). Mechanisms of directed attention in the human extrastriate cortex as revealed by functional MRI. Science 282, 108–111. 10.1126/science.282.5386.108 9756472

[B23] Kastner S. Pinsk M. De Weerd P. Desimone R. Ungerleider L. (1999). Increased activity in human visual cortex during directed attention in the absence of visual stimulation. Neuron 22, 751–761. 10.1016/S0896-6273(00)80734-5 10230795

[B24] Kesner R. P. Rolls E. T. (2015). A computational theory of hippocampal function, and tests of the theory: new developments. Neurosci. Biobehav. Rev. 48, 92–147. 10.1016/j.neubiorev.2014.11.009 25446947

[B25] Miller E. K. Gochin P. M. Gross C. G. (1993). Suppression of visual responses of neurons in inferior temporal cortex of the awake macaque by addition of a second stimulus. Brain Res. 616, 25–29. 10.1016/0006-8993(93)90187-R 8358617

[B26] Motter B. C. (1993). Focal attention produces spatially selective processing in visual cortical areas V1, V2, and V4 in the presence of competing stimuli. J. Neurophysiol. 70, 909–919. 10.1152/jn.1993.70.3.909 8229178

[B27] Panzeri S. Rolls E. T. Battaglia F. Lavis R. (2001). Speed of feedforward and recurrent processing in multilayer networks of integrate-and-fire neurons. Netw. Comput. Neural Syst. 12, 423–440. 10.1088/0954-898X/12/4/302 11762898

[B28] Renart A. Moreno R. Rocha J. Parga N. Rolls E. T. (2001). A model of the IT–PF network in object working memory which includes balanced persistent activity and tuned inhibition. Neurocomputing 38–40, 1525–1531. 10.1016/S0925-2312(01)00548-3

[B29] Renart A. Parga N. Rolls E. T. (1999a). Associative memory properties of multiple cortical modules. Network 10, 237–255. 10.1088/0954-898X_10_3_303 10496475

[B30] Renart A. Parga N. Rolls E. T. (1999b). Backprojections in the cerebral cortex: implications for memory storage. Neural Comput. 11, 1349–1388. 10.1162/089976699300016278 10423499

[B31] Renart A. Parga N. Rolls E. T. (2000). A recurrent model of the interaction between the prefrontal cortex and inferior temporal cortex in delay memory tasks, in Advances in Neural Information Processing Systems, Vol. 12, eds Solla S. Leen T. Mueller K.-R. (Cambridge, MA: MIT Press), 171–177.

[B32] Reynolds J. Desimone R. (1999). The role of neural mechanisms of attention in solving the binding problem. Neuron 24, 19–29. 10.1016/S0896-6273(00)80819-3 10677024

[B33] Reynolds J. H. Chelazzi L. Desimone R. (1999). Competitive mechanisms subserve attention in macaque areas V2 and V4. J. Neurosci. 19, 1736–1753. 10.1523/JNEUROSCI.19-05-01736.1999 10024360 PMC6782185

[B34] Rolls E. T. (2012). Invariant visual object and face recognition: neural and computational bases, and a model, VisNet. Front. Comput. Neurosci. 6:35. 10.3389/fncom.2012.00035 22723777 PMC3378046

[B35] Rolls E. T. (2016). Cerebral Cortex: Principles of Operation. Oxford: Oxford University Press.

[B36] Rolls E. T. (2018). The storage and recall of memories in the hippocampo-cortical system. Cell Tissue Res. 373, 577–604. 10.1007/s00441-017-2744-3 29218403 PMC6132650

[B37] Rolls E. T. Cheng W. Gilson M. Gong W. Deco G. Zac Lo C.-Y. (2019). Beyond the disconnectivity hypothesis of schizophrenia. Cereb. Cortex. 10.1093/cercor/bhz161 31381086

[B38] Rolls E. T. Deco G. (2002). Computational Neuroscience of Vision. Oxford: Oxford University Press.

[B39] Rolls E. T. Deco G. (2010). The Noisy Brain: Stochastic Dynamics as a Principle of Brain Function. Oxford: Oxford University Press. 10.1016/j.pneurobio.2009.01.006 19428958

[B40] Szabo M. Almeida R. Deco G. Stetter M. (2004). Cooperation and biased competition model can explain attentional filtering in the prefrontal cortex. Eur. J. Neurosci. 19, 1969–1977. 10.1111/j.1460-9568.2004.03211.x 15078571

[B41] Treves A. (1993). Mean-field analysis of neuronal spike dynamics. Network 4, 259–284. 10.1088/0954-898X/4/3/002

[B42] Usher M. Niebur E. (1996). Modelling the temporal dynamics of IT neurons in visual search: a mechanism for top-down selective attention. J. Cogn. Neurosci. 8, 311–327. 10.1162/jocn.1996.8.4.311 23971503

